# Mitotic bookmarking by transcription factors

**DOI:** 10.1186/1756-8935-6-6

**Published:** 2013-04-02

**Authors:** Stephan Kadauke, Gerd A Blobel

**Affiliations:** 1 Division of Hematology, The Children’s Hospital of Philadelphia, Philadelphia, PA, 19104, USA; 2 Perelman School of Medicine at the University of Pennsylvania, Philadelphia, PA, 19104, USA

**Keywords:** Mitosis, Bookmarking, Hematopoiesis, Chromatin, Reprogramming

## Abstract

Mitosis is accompanied by dramatic changes in chromatin organization and nuclear architecture. Transcription halts globally and most sequence-specific transcription factors and co-factors are ejected from mitotic chromatin. How then does the cell maintain its transcriptional identity throughout the cell division cycle? It has become clear that not all traces of active transcription and gene repression are erased within mitotic chromatin. Many histone modifications are stable or only partially diminished throughout mitosis. In addition, some sequence-specific DNA binding factors have emerged that remain bound to select sites within mitotic chromatin, raising the possibility that they function to transmit regulatory information through the transcriptionally silent mitotic phase, a concept that has been termed “mitotic bookmarking.” Here we review recent approaches to studying potential bookmarking factors with regards to their mitotic partitioning, and summarize emerging ideas concerning the in vivo functions of mitotically bound nuclear factors.

## Introduction/Overview

Mitosis imposes dramatic and dynamic changes on nuclear organization and gene expression in eukaryotic cells. In metazoans, the nuclear envelope temporarily disintegrates, most nuclear structures are transiently dissolved or rearranged, and nuclear transcription by all three polymerases is globally arrested. Silencing of gene expression is accompanied by the separation of most transcriptional regulators from mitotic chromatin. Following chromosome segregation and re-formation of the nuclear envelope, bulk transcription resumes in the daughter cells that ultimately re-acquire gene expression patterns that are often similar to or indistinguishable from those of the mother cell. It has been widely assumed that these transitions present a problem for the cell’s maintenance of transcriptional identity, prompting investigations into mechanisms that ensure rapid and faithful restoration of gene expression upon re-entry into the G1 phase of the cell cycle. Such mechanisms fall under the category of epigenetics in the stricter sense of the word by providing a cellular memory function throughout the cell division cycle [1]. However, one might question the need for such dedicated mechanisms since the half-lives of most transcripts exceed the duration of mitosis [2]. Therefore, the appropriate regulatory milieu is essentially maintained through mitosis if factors segregate passively in a random fashion. Most protein-DNA contacts are highly dynamic even in interphase (see [3] for review), and consequently, mitotically displaced nuclear regulators would be expected to reload on the correct targets by mass action [4]. This process might be aided by mitotically stable properties of chromatin such as post-translational modifications or nucleosome architecture. However, transcription factors are employed combinatorially at distinct genes and lineages, which allows a limited set of factors to control diverse gene expression programs. This raises the question whether following mitosis, association of these regulators with lineage- or developmental stage-inappropriate genes could lead to changes in cellular growth or differentiation.

Such considerations led to the exploration of diverse mechanisms of gene bookmarking in mitosis to prevent potentially detrimental alterations in gene expression upon re-entry into G1 [5]. These include the retention of DNA binding proteins or transcription co-factors on mitotic chromosomes, mitotically stable histone modifications and histone variants, as well as features of nucleosome architecture and even DNA topology that may at least partially persist through mitosis. Several thorough reviews covered these topics in the recent past [4,6-10].

A different perspective on the effects of mitosis on gene expression comes from the notion that genome-wide perturbations in transcription factor occupancy might facilitate changes in cell fate by allowing the reshaping of transcription programs. An impressive example is the observation that zygotes are capable of reprogramming somatic nuclei only after recipient cells have been arrested in mitosis [11], suggesting that mitosis is required for the release of reprogramming factors from chromatin to reset transcription in donor chromatin. An open question is to what extent transition through mitosis is a more general requirement for establishing lineage diversification. Asymmetrical cell divisions trigger differences in lineage choice of progeny cells or can separate a daughter cell with self-renewal capacity from one that proceeds to differentiate [12,13]. It is possible that in these scenarios newly created transcription environments act on post-mitotic chromatin (as opposed to interphase chromatin) to initiate new transcription patterns in newborn cells. Hence mitosis might be viewed as a window of opportunity for remodeling the transcriptional landscape, which implies that putative bookmarking mechanisms remain sufficiently flexible to permit changes in cellular fate or differentiation.

In this article instead of providing an extension of previous comprehensive reviews of known factors and histone marks that persist on mitotic chromatin, we aim to highlight recent technical and conceptual developments that approach questions of mitotic bookmarking.

## Review

### Methods to study mitotic bookmarking

Here we provide a brief review of frequently used approaches to study potential mitotic memory mechanisms, since inconsistencies or controversies in the literature can be rooted in different methodologies used. Immunofluorescence (IF) microscopy is commonly employed to globally survey mitotic partitioning of nuclear factors and persistence of histone marks. An advantage of IF is that it detects endogenous proteins in their natural context, but it requires suitable antibodies for which the epitope is not occluded by chromatin compaction during mitosis, by mitosis-specific post-translational modifications, or because of fixation. These problems can be sidestepped by live cell-imaging with ectopically expressed fluorophore-tagged molecules. However, overexpression might lead to shifts in mitotic occupancy patterns. The monitoring of histone marks in living cells is more challenging, although strategies have been developed using fluorescence resonance energy transfer (FRET)-based indicator molecules that specifically interact with a given histone mark [14,15]. Given the complexities of histone marks and their molecular interactions, as well as the potential influence of neighboring marks, this approach, although creative, is not without limitations and is not yet universally applicable.

To localize nuclear factors or histone marks on specific genomic sites in mitosis, chromatin immunoprecipitation (ChIP) can be used conventionally or in combination with high throughput sequencing. Concerns about epitope recognition in mitotic cells are similar to those described for IF. The preparation of pure mitotic cells for ChIP is essential and has been aided by the recent development of effective protocols that use antibodies against phosphorylated histone H3 serine 10, a modification globally enriched during mitosis in all cell types, for fluorescence activated cell sorting (FACS) [16,17]. This is especially relevant in cases where cells cannot be easily synchronized by pharmacologic treatments such as nocodozale.

The combinatorial use of IF, live cell imaging, and ChIP does not only serve to corroborate key results, but can also uncover new concepts. For example, the hematopoietic transcription factor GATA1 globally separates from mitotic chromosomes as revealed by IF [16,18]. However, live cell-imaging and genome-wide location analysis by ChIP showed partial retention of this factor [16]. Moreover, ChIP detected significant mitosis-specific shifts in genomic occupancy patterns of the histone methyltransferase MLL, which was not visible by IF [19]. Notably, transcription factor FoxA1 is globally retained on mitotic chromatin as visualized by IF, even though its binding at specific binding sites as measured by ChIP is substantially diminished [20]. These findings suggest the existence of distinct layers of mitotic retention and highlight the importance of combining multiple techniques for the evaluation of potential mitotic bookmarking factors.

Additional strategies to assess mitotic chromatin binding involve the fractionation of mitotic chromosomes followed by western blotting (for example, [21]) or unbiased proteomic analysis [22]. These approaches are suitable to address questions of global chromatin association of one or many factors within a population of cells. As with all biochemical extraction protocols, conditions greatly influence the integrity and purity of large molecular complexes, requiring the implementation of multiple classifiers and independent validation [22].

The methods mentioned so far are useful tools for untangling the protein composition and post-translational modifications of mitotic chromosomes. However, the mere presence of a factor or histone mark on mitotic chromatin is insufficient to establish a functional role in mitotic memory. In several studies select mitotically retained factors were depleted by RNAi, leading to a delay in postmitotic target gene reactivation [19,20,23-26]. However, it is difficult to establish with certainty that the observed effects were solely due to mitotic bookmarking and not to interphase functions of these factors. In recent reports both gain-of-function experiments as well as mitosis-specific loss of function experiments were carried out to address this issue. The acetyl-histone binding protein BRD4 is known to remain on mitotic chromosomes in some cell types [23,27]. Using a stably integrated inducible gene that allows for live monitoring of transcription, the Spector group observed that initial activation of this gene in interphase occurred with much slower kinetics than its re-activation upon mitotic exit [25]. To test whether BRD4 contributes to a potential mitotic transcriptional memory mechanism that accounts for this rapid re-activation, they took two approaches. In the first, they displaced BRD4 from the gene using a competitive inhibitor of acetyl-lysine (JQ1), which delayed postmitotic reactivation but did not affect activation of this gene in interphase. In the second, they tethered BRD4 to the locus, which accelerated transcription induction in interphase. This suggests that BRD4 via association with acetylated chromatin plays a role in transcriptional memory. It will be interesting to examine whether tethering of putative bookmarking factors to non-bookmarked native genes, perhaps via artificial zinc finger proteins or transactivator-like (TAL) factors, can accelerate their postmitotic gene activation.

In an attempt to determine a mitotic bookmarking function for GATA1 Kadauke *et al*. devised a strategy to deplete GATA1 selectively in mitosis [16]. GATA1 was fused to the mitosis-specific degron of cyclin B and stably introduced into GATA1-null cells. Mitosis-specific destruction of GATA1 selectively delayed post-mitotic reactivation of GATA1-bookmarked genes [16]. It should be possible to extend this approach to factors for which knockout cells are unavailable by knockdown-rescue experiments.

Finally, it is worth noting that mitotic partitioning of nuclear factors and histone marks can vary between cell types and might be influenced by whether primary or transformed cells are used (see [28] for review).

### Function

One of the most important questions waiting to be resolved concerns the biological role of the mitotic retention of nuclear factors. Proposed functions include promoting rapid transcriptional activation of mitotic target genes in newborn cells to facilitate the M/G1 transition, as well as the faithful maintenance of lineage choice and differentiation stage. For some factors that were examined for mitotic occupancy on a genome-wide scale, it is notable that the fraction of bound protein and the number of genomic occupied sites on mitotic chromosomes is small when compared to interphase chromatin [16,20,21]. Whether the former are simply remnants of incompletely removed proteins or actually functionally relevant for postmitotic transcriptional regulation is a challenging question that can be tackled with some of the aforementioned gain- and loss-of-function approaches.

In several recent reports depletion of mitotically retained transcription factors delayed transcription reactivation of target genes upon exit from mitosis [16,19,20,23-26]. Several genes expressed in the M/G1 transition but not those expressed at later stages of the cell cycle were found to be stably occupied by BRD4 through mitosis and require BRD4 for their rapid reactivation in telophase [23,24,29], supporting the idea that BRD4 marks these genes to ensure rapid transcription activation and cell cycle progression. However, it is also clear that genes with no known specific roles in G1 progression, such as those encoding lineage-specific nuclear factors, depend on mitotic occupancy by bookmarking factors for their rapid reactivation [16,20,26]. Assessment of the importance of fast transcription reactivation of a given gene also requires knowledge of transcript stability through mitosis. For those transcripts that are stable, transcriptional reactivation kinetics might not be limiting for G1 entry. Therefore, bookmarking of genes producing long-lived transcripts might serve functions other than cell cycle progression.

While timely transcription reactivation has been used as the predominant functional read-out of mitotic bookmarking, it remains unresolved to what extent this is important beyond simply allowing cell cycle progression. It has been proposed that mitotic bookmarking factors may stabilize lineage fidelity by facilitating transmission of the cell type-specific transcriptional state from mother to daughter cells. The retention of select tissue-restricted transcription factors on mitotic chromosomes [16,18,20,30] provides correlative support for such a model. It is possible that factors that remain bound during mitosis at specific targets are prevented from binding to off-target sites, or block competing factors from binding, thus preserving gene expression patterns. The idea that mitosis might be a labile state amenable to global changes in gene expression (such as those associated with cellular reprogramming [11,31,32]) further suggests the existence of mechanisms that counter dramatic changes in gene transcription. Nevertheless, direct evidence that mitotic bookmarking is required for cementing lineage choice is lacking.

Many lineage-determining transcription factors function not only as activators of lineage-affiliated genes but also as repressors of genes associated with alternative lineages or inappropriate maturation stages. Therefore, if mitotic bookmarking is at all relevant to preserving cellular identity, the ability to stably silence gene expression during mitotic exit is likely to be of equal importance. For example, destruction of GATA1 specifically during mitosis was found to lead not only to delayed reactivation of bound genes but to inappropriately high expression of GATA1-repressed target genes [16]. These include *Gata2* and *Kit* that are normally transcribed at an earlier developmental stage but are also expressed in alternative GATA1-dependent hematopoietic lineages, namely mast cells. Together, this suggests but does not prove that GATA1 mitotic bookmarking plays a role in maintaining cellular maturation, lineage fidelity, or both.

The nuclear factor Runx2 is involved in bone formation by activating osteoblast gene expression programs but also functions as a repressor of RNA polymerase 1-transcribed ribosomal genes. Runx2 is focally retained on mitotic chromosomes, including sites of rRNA production [26,33]. Whether Runx2 association with mitotic chromatin is important for rDNA repression or for maintaining the osteoblast lineage, and to what extent this function is mediated by its role as activator versus repressor, remain open questions.

If retention of nuclear factors on mitotic chromatin is indeed important for cementing cell type-specific transcription patterns, we speculate that such bookmarking mechanisms are dynamic throughout development. As an example, multipotent progenitor cells display substantial stochastic cell-to-cell variation in gene expression leading to the seemingly random activation of lineage-affiliated gene expression patterns and even the formation of lineage-committed states in small subpopulations (for example see [34]). It is possible that this transcriptional flux is enabled in part by the lack of mitotic bookmarking mechanisms. Gene expression patterns might be stabilized once commitment is established by nuclear factors capable of repressing alternative lineage affiliated genes. Whether or not mitotic bookmarking serves to resist cellular reprogramming or stochastic changes in gene expression that might lead to lineage infidelity is amenable to investigation. For example, does the disabling of bookmarking factors enhance the efficiency of lineage reprogramming factors to alter cell fate? Are engineered versions of mitotically unstable nuclear factors less efficient in cellular reprogramming than their normal counterparts?

Finally, transcription factors both influence and are influenced by their chromatin environment. The dynamics of histone modifications during mitosis and possible roles in bookmarking are discussed elsewhere [10].

### Mechanisms

Mechanisms that allow dispersal of nuclear factors and modulate histone modifications during mitosis have been extensively studied. Distinct kinetics of dissociation and re-association of factors during mitosis as well as varying stabilities of histone marks argue that multiple mechanisms control mitotic partitioning. Nevertheless, common themes have emerged that apply to broad classes of molecules, including dynamic phosphorylation of histones and nuclear factors. We will not discuss these mechanisms here as they have been thoughtfully covered in several recent reviews [4,10,28]. Instead, we will discuss the related but distinct questions of how select factors are able to remain associated with chromatin at some sites but not others, and how their selective retention might influence transcriptional reactivation or contribute to the maintenance of transcription patterns.

Although global occupancy of transcription factors is sometimes reduced during mitosis [16,19-21], it is notable that mitotic Runx2 binding intensities seem to match those of interphase cells at numerous sites although this has not yet been examined on a genome-wide scale [26]. In the cases of MLL1 and FoxA1 [19,20] but not GATA1 [16], mitotic retention favors genes highly expressed in interphase, suggesting that chromatin features and/or transcription factor environment of highly active genes promotes mitotic retention. It is possible that in these scenarios, mitotic bookmarking facilitates the dramatic transitions from off- to on-states when exiting mitosis.

The observed transcription factor occupancy patterns typically fall into distinct classes. At some sites nuclear factors are retained at normal levels while at others binding is diminished, which might be a result of fewer molecules per occupied site or fewer alleles being bound in heterogenous cell populations. In addition, new sites might be occupied by nuclear factors only in mitosis, thus signaling a shift in binding properties [16,19,35]. While the defining features of these groups are still unclear, some ideas are emerging as discussed in the following paragraphs.

#### DNA sequence context

DNA-binding factors generally occupy sites in a sequence-specific fashion, and variation in sequence can affect binding dynamics. Comparative genome-wide occupancy profiles of sequence-specific transcription factors in interphase and mitosis are now beginning to be determined [16,20,21]. DNA binding motif analysis of GATA1 and FoxA1 has not revealed any features that predict whether the factor remains bound or dissociates during mitosis [16,20]. More factors need to be studied to determine whether this is universally true.

An emerging theme of mitosis-specific occupancy involves repetitive DNA sequence elements. During mitosis the *D. melanogaster* GAGA transcription factor shifts from its interphase binding sites to centromeric alpha-satellite GA repeats [35,36]. Moreover, Runx2 concentrates on rDNA repeats in mitosis, which contain a high density of Runx2 binding motifs [33]. The majority of mitosis-only GATA1 target sites tend to localize to (GATA)_n_ repeats in intergenic regions remote from any annotated genes [16]. Why would DNA-binding factors dissociate from their interphase binding sites and occupy repeat sequences? Transcription factor occupancy is influenced by the presence of neighboring nuclear factors that might convey binding synergy, or facilitate binding by association with chromatin modifying factors. Perturbation of transcription factor/co-factor context during mitosis might trigger the dispersal of these factors away from interphase binding sites, making them available for otherwise less favorable locations. This equilibrium is likely influenced by the number of repeat elements. Moreover, the chromatin context of those sites might not be as tightly controlled. It remains unclear what function these mitosis-only binding events serve. In the case of GAGA factor, it is possible that it contributes to packaging of chromatin encompassing repeat sequences during mitotic chromosome condensation [35]. It is also conceivable that these repeat elements serve a transient depot function by restricting movement away from chromatin and allowing more rapid re-association with interphase targets.

#### Local chromatin context

Several features of chromatin such as DNase hypersensitivity, various modifications of DNA and histones, and histone variants persist at least in part through mitosis while others are dynamic throughout the cell cycle [37-47]. It seems reasonable to propose that the chromatin environment might modulate mitotic transcription factor binding. However, at present there are no known features of chromatin that predict with certainty whether a transcription factor remains bound to mitotic chromatin or not. DNase hypersensitive sites are generally maintained in mitotic chromatin [16,37,47] with some exceptions [48]. Although GATA1 contributes to the formation of DNase hypersensitive sites, they remain hypersensitive during mitosis regardless of whether GATA1 persists there or not [16], implicating additional epigenetic mechanisms that maintain them.

However, some features of chromatin structure influence transcription factor binding profiles during mitosis. FoxA1 binding in mitosis seems to favor sites of high nucleosome density, consistent with its strong affinity for nucleosomes [20]. It has also been observed that positioned nucleosomes are lost at some genes during mitosis, raising the possibility that altered nucleosome position impacts on mitotic transcription factor binding or vice-versa [49]. The histone variant H2A.Z, which is associated with active and poised promoters, is retained during mitosis despite transcriptional silencing, although its acetylation is diminished [50]. Interestingly, global analyses revealed that the H2A.Z-containing +1 nucleosome slides towards the 5’ end of genes to occupy transcriptional start sites and shrink the nucleosome-free region at gene promoters [45], potentially providing a universal mechanism for temporary gene silencing. It is possible that repositioning of promoter proximal nucleosomes contributes to the mitotic displacement of basal transcription factors. Investigation into how reversible nucleosome sliding is controlled in such a temporally and spatially specific manner will likely provide important new insights into mitotic chromatin dynamics.

The post-translational histone modifications examined so far fail to clearly distinguish interphase-only from persistent transcription factor binding sites [16,20], although subtle trends do exist. Runx2 mitotic binding is associated with increased H3K4 dimethylation [26]. GATA1 sites that are selectively occupied in mitosis tend to be enriched for the repressive histone marks H3K27me3 and H3K9me3 [16]. However, it is unclear whether this is simply a consequence of the presence of these marks at (GATA)_n_ repeats (see previous section).

The polycomb protein PSC is partially maintained on chromatin in mitosis but not at the prototypic PSC target *Hox* locus, even though the polycomb target mark H3K27me3 is present at this locus throughout mitosis [21]. Notably, almost half of the retained sites fall on boundaries of topological domains that are also occupied by molecules involved in higher order chromatin organization. It will be interesting to examine the local chromatin context of mitotically persistent PSC sites as well as the possible role of PSC bookmarking in the restoration of long-range chromatin contacts after mitosis.

Dynamic phosphorylation of histones, transcription factors, and the mitotic condensation machinery control transitions through mitosis [6,28], and several reports describe mechanisms by which these histone modifications can eject chromatin binding factors during mitosis [10,51,52]. The basal transcription factor TBP, which occupies a subset of promoters in mitosis and HSF2 can interact with the protein phosphatase PP2A, which can dephosphorylate subunits of the condensin complex [53,54]. This leads to the tantalizing proposition that localized dephosphorylation of condensin antagonizes chromatin compaction at select sites leaving a mitotic bookmark [54]. To what extent this mechanism is broadly operational *in vivo* will be an important question for the future. It is notable that PP2A can also reverse the mitotic inactivation of the SWI/SNF chromatin remodeling complex [55], but since SWI/SNF is globally separated from mitotic chromatin it is unclear whether this mechanism functions to antagonize mitotic nucleosome repositioning, unless the complex is retained locally at bookmarked regions.

Acetylation of histones is at least partially maintained during mitosis [7]. Readers of histone acetylation of the BET family proteins BRD2 and BRD4 remain globally associated with mitotic chromosomes in some cell types in a manner dependent on histone acetylation [23,27]. In other cell types BRD4 was found to separate from mitotic chromatin but to re-associate rapidly after mitosis, prior to other examined factors [23,25] (see also above). Potential mechanisms by which BET family proteins act to promote rapid transcriptional reactivation include recruitment of a variety of transcription complexes including P-TEFb, or possibly its intrinsic RNA polymerase 2 kinase activity [23,24,56,57]. Compounds targeting BET family proteins have garnered a great deal of attention for their potential as anti-inflammatory and anti-cancer drugs (see [58] for review). It is possible that some of their activities relate to disruption of mitotic memory functions required to sustain tumor cell growth or the expansion and function of immunocompetent cells.

#### Transcription co-factors

Transcription co-regulators are typically loaded onto chromatin via DNA binding proteins. It is therefore expected that mitotic partitioning of the former mirrors that of the latter. Correspondingly, the majority of cofactors are separated from mitotic chromosomes (Table 1). A notable exception is the histone methyltransferase MLL1, which is globally retained on mitotic chromosomes along with its partners Menin, ASH2L, and RbBP5 (Table 2, [19]). The Runx2 co-repressor TLE1 appears to co-localize with Runx2 on select sites in mitotic chromatin while HDAC1 does not [59], suggesting selectivity among mitotically stable protein interactions. The GATA1 cofactors FOG1 and components of the TAL1 complex dissociate from all binding sites irrespective of whether GATA1 remains or not [16]. This suggests that protein-protein interactions are also dynamic throughout mitosis and that mitotically persistent transcription factors can function as a platform on which cofactor complexes are reassembled, thereby accelerating transcription reactivation.

**Table 1:** Factors that tend to dissociate from mitotic chromosomes

**Basal transcription machinery**	**Sequence-specific transcription factors**	**Transcription cofactors**	**Chromatin remodelers**	**Chromatin writers**	**Chromatin readers**	**Other chromatin proteins**
**POLR2A** (RNApol2) [19,25,43,60-62]	MYC^*^[20,63,64]	**EED** (Esc) [65]	**SMARCA2** (BRM) [66]	**CREBBP** (CBP) [27,43,46,67]	CBX5^*^ (HP1α) [68-72]	**ORC1**[73,74]
**POLR3A** (RNApol3) [75]	**MYB**[47]	**PHC1** (Polyhomeotic) [76,77]	**SMARCA4** (BRG1) [43,66]	EP300^*^ (p300) [30,46]	CBX1^*^ (HP1β) [68,69,71,78]	**SRSF2** (SC-35) [30]
**GTF2A1** (TFIIA) [79]	**FOS**[47,80]	**PCGF6** (MBLR) [85]	**SMARCA5** (SNF2) [43]	**KAT2B** (PCAF) [46]	CBX3^*^ (HP1γ) [68,69,71,78]	**SMC3** (Cohesin) [81]
**GTF2B** (TFIIB) [23,61,62,79,82]	**POU2F1** (OCT1) [47,75,79,83,84]	**ZFPM1** (FOG1) [16]	**CHAF1A** (CAF-1) [68]	**KAT8** (MYST1) [46]		**SCC1** (Cohesin) [86]
**TAF1** (TAF_II_250) [46]	**POU2F2** (OCT2) [47]	**LMO2**[16]	**HIRA**[87]	**NCOA3** (ACTR) [46]		
**TAF3** (TAF_II_140) [52]	MYOD1^*^ (MyoD) [88,89]	**LDB1**[16]		**ELP3**[43]		
**TAF4** (TAF_II_130/135) [90]	**HSF1**[47]			**HDAC1**[46,59]		
TAF5^*^ (TAF_II_100) [52,61]	**E2F1**[47]			**HDAC2**[46,91]		
**TAF12** (TAF_II_20/15) [79]	**BCL6**[47]			**HDAC3**[46]		
**GTF2E1** (TF_II_E p56) [62]	**ETS1**[47]			**HDAC4**[46]		
**GTF2F1** (TFIIF) [23,62]	**SP1**[27,47,84,91]			**HDAC5**[46]		
**ERCC2** (TFIIH XPD) [62]	**SP3**[91]			**HDAC7**[46]		
**ERCC3** (TFIIH XPE) [62]	IKZF1^*^ (Ikaros) [92,93]			**SETD1A**[19]		
BDP1^*^ (TFIIIB) [75]	**PAX3**[94]			**MLL2**[19]		
**TRF2**[90]	**PAX5**[67]			**ASH1L**[19]		
**MED14** (DRIP150) [62]	**HNF4A** (HNF4-α) [95]			**DOT1L**[19]		
**MED23** (DRIP130) [62]	**TBX2**[96]			**KDM1A** (LSD1) [19]		
**CDK9**[23,24]	**ESR1** (ERα) [91]			**RNF2** (RING1B) [77]		
**CCNT1** (Cyclin-T1) [23,24]	**TAL1** (SCL) [16]					
**SSRP1** (FACT) [43]	CTCF^*^[48,97,98]					

Factors that have been reported to be released from mitotic chromosomes are shown and references are provided. For the sake of simplicity, canonical human gene identifiers are shown (boldface) even though data were collected from experiments carried out in human, mouse, frog, insect, worm, and plant cells. For some factors, commonly known names are shown in parentheses. ^*^Indicates conflicting reports, which may be due to differences in species, cell type, and/or techniques by which the factor was examined.

**Table 2:** Factors that are at least partially bound to mitotic chromosomes

**Basal transcription machinery**	**Sequence-specific transcription factors**	**Transcription cofactors**	**Chromatin writers**	**Chromatin readers**
TBP^*^[19,49,54,61,75,79,82,90]	**SRF** (p67) [99]	**MEN1** (Menin) [19]	MLL^*^[19,100]	**L3MBTL1** (L(3)mbt)
**BRF1** (TFIIIB) [75]	**TFAP2A** (AP-2) [47]	**RBBP5**[19]	**ASH2L**[19]	[101]
**TFIIIC2**[75]	**HSF2**[53]	BMI1^*^ (Psc) [21,76,101-103]	**RING1**[21,102]	**BRD2**[104]
	**NFE2** (p45) [18]	TLE1^*^ (Groucho) [59,105]	SUV39H1^*^[106,107]	BRD4^*^[23-25,27,104]
	**RUNX2**[26,30,33,59]	HMGB1^*^ (HMG1) [20,108,109]		CBX8^*^ (Polycomb) [21,76]
	**HNF1B** (HNF1-β) [95]			**CBX4** (Pc2) [102]
	**FOXI1**[110]			
	**FOXA1**[20]			
	GATA1* [16,18]			
	**GATA4**[20]			
	CEBPA^*^ (C/EBP α) [20,47]			
	**CEBPB** (C/EBP β) [111]			
	**UBTF** (UBF-1) [33]			
	**DR1** (NC2) [61]			
	*Dm***Trl** (GAGA factor) [35,112]			
	*Dm***Psq** (Pipsqueak) [112]			
	*At***VRN1**[113]			

Factors that have been reported to remain bound to mitotic chromosomes are shown and references are provided as in Table 1. Factors that lack human homologues are indicated by the species-specific gene name (*Dm*, *Drosophila melanogaster*; *At*, *Arabidopsis thaliana*). ^*^Indicates conflicting reports, which may be due to differences in species, cell type, and/or techniques by which the factor was examined.

## Conclusion

For over half a century it has been known that transcription is globally silenced during mitosis [114,115], yet how the cell copes with the challenges imposed by mitotic reorganization of the genome and nuclear structures is still largely a mystery. The development of new tools, including high-throughput technologies has brought new insights into this question. Thus, histone modifications, nucleosome architecture, and transcription factor-binding during mitosis are beginning to be unraveled. It has become clear that multiple features that discern active from inactive genes are stable through mitosis. Further studies involving diverse cellular systems are required to address similarities, but also distinctions among potential bookmarking mechanisms between lineages and organisms. It will also be important to examine as yet mostly unstudied aspects of gene expression during mitosis, including the fate of non-coding RNAs, or the dynamics of higher order chromatin folding.

To what extent mitotically stable features of chromatin are required for the propagation of transcriptional information and maintenance is still mostly subject to speculation but has moved closer within our reach. Important related questions to be addressed include whether there is a direct role for mitosis in facilitating changes in transcription patterns to allow cell fate decisions during development, and whether alleviation of bookmarking facilitates cellular reprogramming or lineage switching. Finally, it will be important to explore whether perturbation of mitotic bookmarking accounts for developmental disorders or malignancies.

## Abbreviations

ASH2L: Absent, small, or homeotic-like protein; BET: bromodomain-ET; BRD: bromodomain-containing protein; ChIP: chromatin immunoprecipitation; FACS: fluorescence-activated cell sorting; FoxA1: forkhead box A1; FOG 1: Friend of GATA 1; FRET: fluorescence resonance energy transfer; GATA1: GATA binding factor 1; HDAC1: Histone deacetylase 1; IF: immunofluorescence; P-TEFb: positive transcription elongation factor b; PSC: posterior sex combs; RbBP5: Retinoblastoma-binding protein 5; SWI/SNF: switch/sucrose nonfermentable; TAL: transactivator-like; TAL1: T-cell acute lymphocytic leukemia protein 1; TBP: TATA-binding protein; TLE: Transducin-like enhancer protein 1

## Competing interests

The authors declare that they have no competing interests.

## Authors’ contributions

SK and GAB drafted and edited the manuscript. Both authors have read and approved the final manuscript.
